# Optimizing Photoperiod, Exposure Time, and Host-to-Parasitoid Ratio for Mass-Rearing of *Telenomus remus*, an Egg Parasitoid of *Spodoptera frugiperda*, on *Spodoptera litura* Eggs

**DOI:** 10.3390/insects12121050

**Published:** 2021-11-24

**Authors:** Wanbin Chen, Qingfen Weng, Rui Nie, Hongzhi Zhang, Xiaoyu Jing, Mengqing Wang, Yuyan Li, Jianjun Mao, Lisheng Zhang

**Affiliations:** 1 State Key Laboratory for Biology of Plant Diseases and Insect Pests, Institute of Plant Protection, Chinese Academy of Agricultural Sciences, Beijing 100193, China; chenwb24@126.com (W.C.); jingxiaoyu1996@163.com (X.J.); wangmengqing@caas.cn (M.W.); liyuyan@caas.cn (Y.L.); jianjunmao@ippcaas.cn (J.M.); 2 College of Plant Protection, Henan Agricultural University, Zhengzhou 450002, China; 17337619318@163.com (Q.W.); nierui0721@163.com (R.N.); 3 Department of Entomology and BIO5 Institute, University of Arizona, Tucson, AZ 85721, USA; zhanghz@email.arizona.edu

**Keywords:** mass-rearing efficiency, biological control, host egg-to-parasitoid ratio, photoperiod, exposure time, production costs

## Abstract

**Simple Summary:**

*Telenomus remus* (Nixon) is a promising natural enemy of *Spodoptera frugiperda* (J. E. Smith). Successful implementation of a biocontrol program requires a mature rearing system to produce millions of beneficial insect products at lower costs. This parasitoid is successfully reared on *Corcyra cephalonica* (Stainton) eggs in several countries, however that host species is unsuitable for Chinese strains of *T. remus*. Fewer studies have been done using *Spodoptera litura* (Fabricius) eggs, but it is increasingly seen as the promising alternative host in China. In order to identify optimal mass-rearing conditions when using *S. litura* eggs as an alternative host, this novel study thus sought to comprehensively evaluate the effects of photoperiod, exposure time, and host egg:parasitoid ratio on the reproductive potential and mass-rearing efficiency of *T. remus* on *S. litura* eggs. Our results suggest using more than 12 h of light, 24 h exposure time, and 14–20:1 host egg:parasitoid ratio for rearing *T. remus* on *S. litura* eggs. These findings will help promote successful, large-scale rearing of *T. remus* for use against *S. frugiperda* in China.

**Abstract:**

*Telenomus remus* (Nixon) is a dominant egg parasitoid of the destructive agricultural pest *Spodoptera frugiperda* (J. E. Smith)*,* and so is used in augmentative biocontrol programs in several countries. An optimized mass-rearing system is essential to produce biological control products in a timely and cost-effective manner. In this study, the photoperiod, host egg:parasitoid ratio, and exposure time were evaluated to identify the optimal rearing conditions for *T. remus* on the alternative host *Spodoptera litura* (Fabricius) eggs. Results showed that increasing photoperiod above 12L:12D remarkably improved parasitoid progeny yield and life table parameters. Overlong photoperiods shortened female longevity, but within acceptable limits. There was a significant negative correlation between parasitism rate and host egg:parasitoid ratio under exposure times of 12 and 36 h, but not 24 h. Percentage of female progeny increased significantly along with increasing the host egg:parasitoid ratio. A significant negative relationship between the number of emerged adults per egg and the host egg:parasitoid ratio was observed at an exposure time of 36 h. It was concluded that *T. remus* may be mass-reared most efficiently on *S. litura* eggs using a photoperiod of more than 12L:12D, a 14–20:1 host egg:parasitoid ratio, and an exposure time of 24 h. These findings can be used to produce *T. remus* more efficiently and at lower costs.

## 1. Introduction

*Spodoptera frugiperda* (J. E. Smith) is an indigenous species in America [1], but has spread to 44 African countries [2] and several Asian countries [3] in less than three years since it first invaded Africa in 2016 [4]. Field investigations first detected it in China in January 2019 [5]. By September 2020, it had spread to 27 provinces (autonomous regions and municipalities) across the country [6]. Such invasive insects have considerable negative influences on their new environments, damaging indigenous plant species and becoming major agricultural pests [7]. The economic losses caused by invasive insects, in general, are estimated to be about US$1.3 trillion per year worldwide [8], and *S. frugiperda* is widely agreed to be one of the major devastating invasive pests [3,9,10]. Its larvae are highly polyphagous with over 353 recorded host plants, such as corn, wheat, rice, cotton, potato, onion, and sorghum [11]. It poses a particular threat to corn production in many countries, which is the third most cultivated grain in the world [12,13]. In Africa, this pest has been calculated to cause annual yield losses in corn of 21–53%, with the financial losses estimated at approximately US$2.48–6.19 billion [9].

At present, synthetic chemical pesticides are still the most common and effective emergency method for *S. frugiperda* control [14]. Predictably, long-term pesticide application comes with insecticide resistance, secondary pest outbreaks, elimination of natural enemies, and potential environmental and human health challenges [15,16]. With the realization of adverse effects associated with the application of chemical pesticides, agriculture is gradually transforming to more eco-friendly and sustainable pest control techniques [17]. The biological control of insect pests is a vital component of integrated pest management, and provides a self-sustaining strategy for the management of alien invasive pests [7]. Correspondingly, the Chinese government has launched a series of research programs to advocate the reduced application of conventional pesticides and support the development of sustainable agriculture [15]. Therefore, it is necessary to develop an ecologically safe and sustainable strategy for the control of *S. frugiperda*.

*Telenomus remus* (Nixon) is a promising egg parasitoid widely applied in the Americas to control *S. frugiperda* [18,19,20]. In Honduras, releasing 35,000–105,000 *T. remus* wasps/ha/week resulted in 20% to 92% of eggs in maize and sorghum being parasitized [19]. In Brazil, the maximal parasitism rate after *T. remus* release at phenological stages V_4_ and V_10_ in corn, vegetative, and reproductive stages in cotton, and vegetative and reproductive stages in soybean fields respectively reached 99.8% and 96.8%, 77.8% and 73.1%, and 77.3% and 54.4% [21]. Such success is due in large part to its excellent ability to overcome the scales covering *S. frugiperda* egg masses and parasitize the eggs arranged in the inner layer [19,22].

Successful implementation of a biological control program requires a well-developed rearing system that can mass-produce millions of beneficial insect products while efficiently using resources, space, and time [15,23]. Production efficiency and cost are thus key issues affecting the development of the biological control industry [24].

The identification of a suitable host is necessary to develop a parasitoid mass-rearing system, which will be optimized specifically for that parasitoid-host interaction. Several reports have studied the ideal rearing methods and conditions for *T. remus* on *Corcyra cephalonica* (Stainton) eggs [25,26,27,28], a feasible host in certain parts of the world. In addition, eggs of *Agrotis biconica* (Kollar), *A. ipsilon* (Hufnagel), *S. exigua* (Hübner), *S. littoralis* (Boisduval), and other Lepidoptera species have also been served as hosts [19]. However, Chinese studies found that the indigenous population of *T. remus* cannot parasitize *C. cephalonica* eggs, making this host unsuitable in China [29,30]. The authors speculated that this may be because wild-caught *T. remus* bred on the natural hosts cannot recognize and parasitize the *C. cephalonica* eggs after being taken into a laboratory [30]. Chinese reports have suggested *Spodoptera litura* (Fabricius) eggs as a candidate host. Our prior study evaluated the biological parameters of *T. remus* reared on *S. litura* eggs, such as development, thermal requirement, parasitism, and offspring fitness, and the results verified the feasibility of this alternative host [31]. The next step, in order to reduce production costs, is to do a series of tests to carefully assess the effects of different factors on mass production efficiency in the environment of a laboratory [32] and eventually identify the optimal mass-rearing conditions.

Among several such factors, exposure time and the host egg:parasitoid ratio impact the reproductive efficiency of parasitoids in mass production systems [32,33,34]. Several studies focused on the effects of these two factors on other parasitoids, including *Sclerodermus pupariae* (Yang & Yao) [34], *Ontsira mellipes* (Ashmead) [33], and *Trichogramma* [24], and found that unsuitable times and ratios can reduce rearing efficiency to different degrees. For example, although approximately 50–260 *Trichogramma dendrolimi* (Matsumura) can emerge from one *Antheraea pernyi* (Guérin-Mèneville) egg, the offspring body size, female ratio, longevity, and reproductive capacity will decrease if too many adults emerge from each egg [35]. To avoid degeneration and superparasitism, it is essential to identify a suitable exposure time and host egg:parasitoid ratio during mass production; in the above system, the optimal host egg:parasitoid ratio is 1:2, and the optimal exposure time is less than 24 h [35]. In addition, it is necessary to study the impact of abiotic factors on the biological characteristics of parasitoids to maximize mass production efficiency [36]. Although the role of temperature [31,37] and relative humidity [28,38] on parasitism and development are known, there is a gap in knowledge about the effect of photoperiod on the biology of *T. remus* on *S. litura* eggs.

Therefore, in order to achieve the best production levels that satisfy the needs from the field at lower costs, this study aimed to measure the effect of photoperiod, exposure time, and the host egg:parasitoid ratio on the parasitism rate, emergence rate, percentage of female progeny, and other biological parameters of *T. remus* reared on *S. litura* eggs.

## 2. Materials and Methods

### 2.1. Insects

The larvae of *S. frugiperda* were collected in 2019 from Kunming, Yunnan Province, China. The first to third instar young larvae were reared together in rectangular containers (34 cm length × 22 cm width × 4 cm height) and provided with fresh corn leaves. While fourth to sixth instar old larvae were reared individually by an artificial diet [39] in cylindrical boxes (3 cm height × 5 cm diameter). The component and preparation of the artificial diet were referred to those described by Greene et al. [39] with a slight modification; the pinto beans were replaced with soybean powder. Once adults emerged, they were placed in cylindrical wire-mesh cages (24 cm diameter × 28 cm height) with wet gauze and wax paper as oviposition substrates and 20% honey solution provided as food. The substrates with egg masses were collected daily for subsequent experimentation. The population was maintained in a climatic incubator at 28 ± 1 °C, 60 ± 5% relative humidity (RH), and 16:8 Light (L):Dark (D). All the climatic incubators (RXZ-500) used in this study were manufactured by the Ningbo Jiangnan Instrument Factory, Zhejiang Province, China.

Eggs of *S. litura* were originally purchased from the Jilin Academy of Agricultural Sciences, Jilin Province, China. The artificial diet for raising *S. litura* was prepared according to the formula reported by Chen et al. [40]. The feeding technology and conditions were the same as those of *S. frugiperda* as mentioned above. In order to ensure the availability of experimental materials, we continuously and large-scale raised the population.

*T. remus* were obtained from a colony maintained in the corn pest laboratory at the Institute of Plant Protection, Chinese Academy of Agricultural Sciences, in Beijing. The founder specimens were originally collected in 2019 from Jinhua, Zhejiang Province, China. Adult parasitoids were released in plastic tubes (10 cm height × 2.5 cm diameter) containing egg masses of the host *S. frugiperda* and fed with 20% honey solution under 26 ± 1 °C, 70 ± 5% RH, and 14L:10D.

### 2.2. Parasitism and Fitness of T. remus under Different Photoperiods

The following seven photoperiods (L:D) were tested: 0:24, 4:20, 8:16, 12:12, 16:8, 20:4, and 24:0. A single mated female parasitoid emerged within 24 h and was introduced into a plastic tube (10 cm height × 2.5 cm diameter) containing a drop of 20% honey solution in the inner surface, and then one paper card with approximately 160 *S. litura* eggs (based on the known daily parasitism rate of *T. remus* on *S. litura* eggs [31]) was placed into the tube. All trials were conducted in the same temperature and relative humidity as mentioned above for *T. remus* rearing, differing only in the photoperiod. The female parasitoid was checked daily to measure her longevity, and the egg card was refreshed daily until her death. The parasitized eggs were moved into a new tube and maintained at the same conditions until emergence. Any *S. litura* larvae hatched from unparasitized eggs were removed in a timely manner to keep them from eating other eggs. The observation of parasitism and fitness was repeated five times, with each five wasps as a replicate. The total number of parasitized eggs/female and the emergence rate and percentage of female progeny were recorded.

### 2.3. Effects of Exposure Time and Host Density

To identify the optimal host egg:parasitoid ratio, and optimal duration of exposure to parasitoids, the completely randomized design had two factors: Exposure time with three levels (12, 24, and 36 h) and host egg:parasitoid ratio with 11 levels (1:1, 2:1, 4:1, 6:1, 8:1, 10:1, 12:1, 14:1, 16:1, 18:1, and 20:1), for a total of 33 treatments. Approximately 80 mated female parasitoids within 24 h since emergence were released in tubes containing *S. litura* eggs (less than 24 h of age) based on the ratio described above. Therefore, the corresponding host densities were 80, 160, 320, 480, 640, 800, 960, 1120, 1280, 1440, and 1600 eggs. The parasitism conditions were maintained in 26 ± 1 °C and 70 ± 5% RH, which are the optimal conditions as identified in our previous study [31]. However, the photoperiod was set as 24 h light, as *T. remus* is mostly inactive in the dark based on above photoperiod trials, and the phenomenon was also observed while maintaining the population. After the set exposure time, the parasitized eggs were removed into a new tube for development. Once offspring emerged, the number of female and male parasitoids and number of parasitized eggs were recorded. All treatments were replicated five times.

### 2.4. Statistical Analysis

One-way Analysis of Variance (ANOVA) was employed to determine the effects of photoperiod on the total number of parasitized eggs, longevity, emergence rate, and percentage of female progeny, and the means were compared using Tukey’s honest significant difference (HSD) test at a 0.05 significance level. Life table parameters of the intrinsic rate of increase (*r_m_*), finite rate of increase (*λ*), net reproductive rate (*R_0_*), and the mean generation time (*T*) for each photoperiod were estimated using the methods mentioned by Huang et al. [41].

The interactive effects between exposure time and host egg:parasitoid ratio on parasitism rate, emergence rate, percentage of female progeny, and the number of emerged adults per egg were analyzed by two-way ANOVA. The relationship between the biological parameters mentioned above and host egg:parasitoid ratio was fitted by linear regression. All percentages were arcsine square-root-transformed to homogenize the variances before analysis, while the data for the number of parasitized eggs, longevity, and number of emerged adults per egg were log 10-transformed. All analyses were carried out in SPSS version 19.0 (IBM Corp., Chicago, IL, USA), and the figures were drawn in GraphPad Prism version 8.0 (GraphPad Software, Inc., San Diego, CA, USA).

## 3. Results

### 3.1. Effect of Photoperiod on Parasitoid Performance

**Figure 1:**
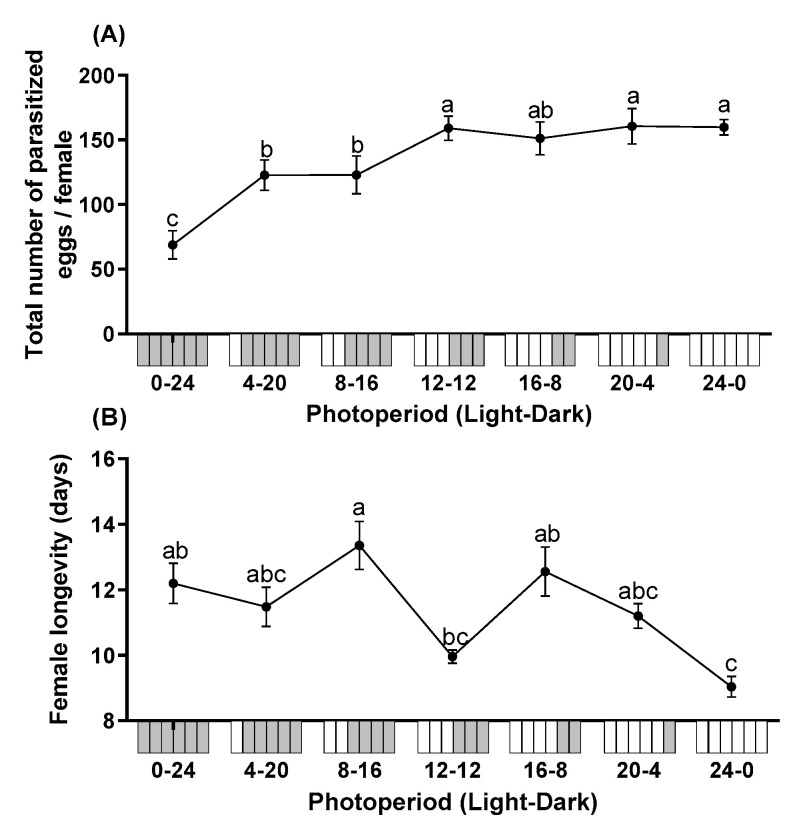
The total number of parasitized eggs per female (**A**) and longevity (**B**) of *T. remus* using *S. litura* eggs as alternative host under the different photoperiods. Data are represented as mean ± SE. Different lowercase letters indicate significant differences among several photoperiods at α = 0.05 (Tukey test).

The total number of parasitized eggs differed significantly among different photoperiods (F = 36.315; df = 6, 28; *p* < 0.0001). Mean fecundity of *T. remus* under constant dark conditions (0L:24D) was lowest, which was significantly lower than all photoperiods tested in this study. Overall, the total number of parasitized eggs increased with longer photoperiods (Figure 1A). The photoperiod also had significant impacts on female longevity (F = 5.960; df = 6, 28; *p* < 0.0001). The longevity ranged from 9.0 to 13.4 days. The longevity of females reared under constant illumination (24L:0D) was significantly shorter than under photoperiods of 0:24, 8:16, or 16:8 L:D (Figure 1B).

**Figure 2:**
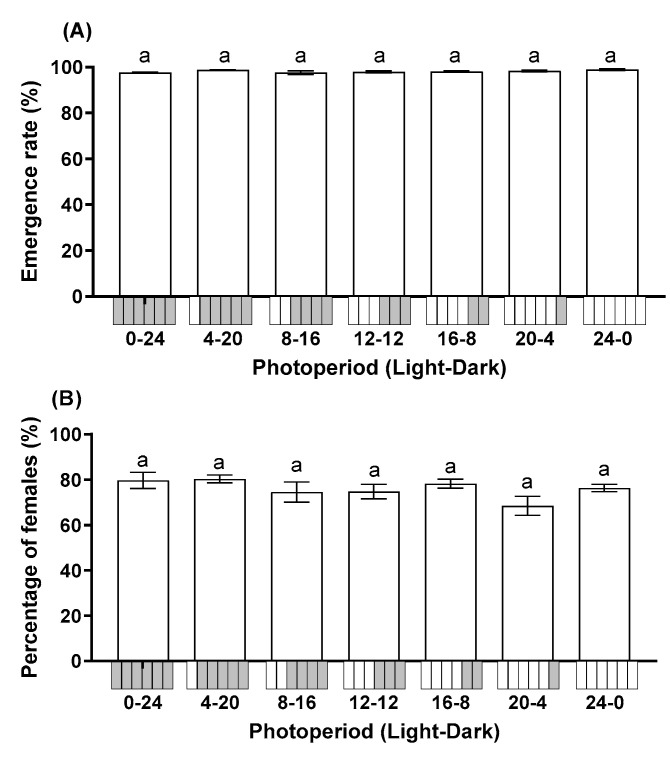
Influence of photoperiod on the emergence rate (**A**) and percentage of female progeny (**B**) of *T. remus* using *S. litura* eggs as the alternative host. Data are represented as mean ± SE. Different lowercase letters indicate significant differences among several photoperiods at α = 0.05 (Tukey test).

Photoperiod did not significantly affect the emergence rate (F = 1.908; df = 6, 28; *p* = 0.115) or percentage of female progeny (F = 1.572; df = 6, 28; *p* = 0.192). The emergence rate across all treatments ranged from 97.57% to 98.90% (Figure 2A). The progeny of *T. remus* was female-biased in all photoperiods with >68.50% of progeny being female (Figure 2B).

### 3.2. Life table of T. remus under Different Photoperiods

**Figure 3:**
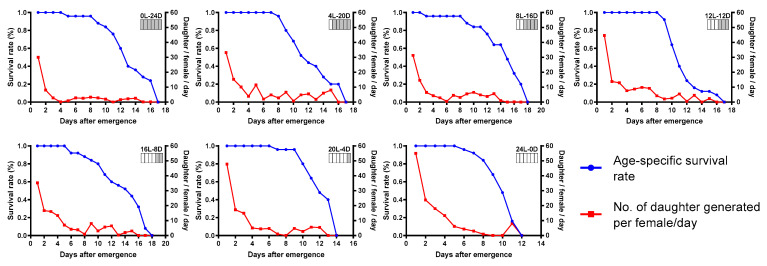
Age-specific survival rate of *T. remus* and number of daughters generated per female per day under different photoperiods.

Life table parameter statistics found that the photoperiod of 24L:0D was preferable compared with other photoperiods tested. As a result of shortened mean generation time and higher parasitism potential, *T. remus* reared with constant light had the highest net reproductive rate, intrinsic rate of increase, and finite rate of increase. The worst performance of *T. remus* was observed in the photoperiod of 0L:24D (Table 1). Age-specific survival rate and the number of daughters generated per female of *T. remus* under different tested photoperiods are illustrated in Figure 3. The number of daughter/female/day was highest on the first day under all of the tested photoperiods. The survival rate of females began to decrease strongly, approximately eight days post-emergence.

### 3.3. Parasitism Rate of T. remus under Different Combinations of Exposure Time and Host Egg:Parasitoid Ratio

**Figure 4:**
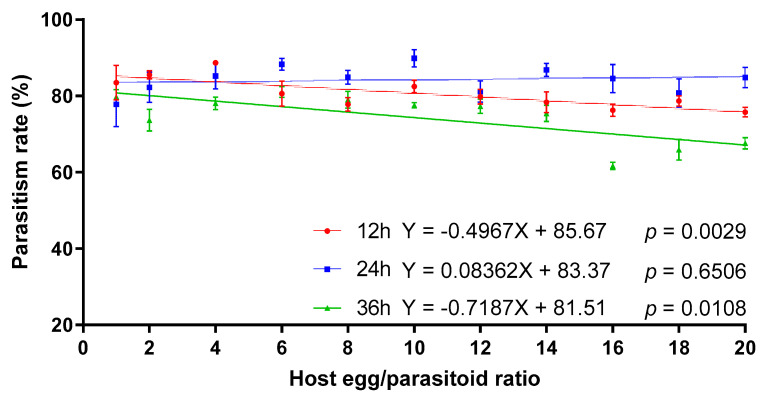
The effect of host egg:parasitoid ratio on parasitism rates of *T. remus* on *S. litura* eggs at different exposure times.

*Telenomus remus* parasitism on *S. litura* eggs was significantly influenced by the interactions between exposure time and the host egg:parasitoid ratio (Table 2). The linear regression indicated that the parasitism rate of *T. remus* on *S. litura* eggs decreased significantly with the increase of the host egg/parasitoid ratio for 12 and 36 h of exposure time. However, there was no significant linear relationship between the parasitism rate and host egg:parasitoid ratio at 24 h of exposure time, and the average parasitism rate ranged from 77.75% to 89.85% (Figure 4).

### 3.4. Offspring Fitness of T. remus under Different Combinations of Exposure Time and Host Egg:Parasitoid Ratio

**Figure 5:**
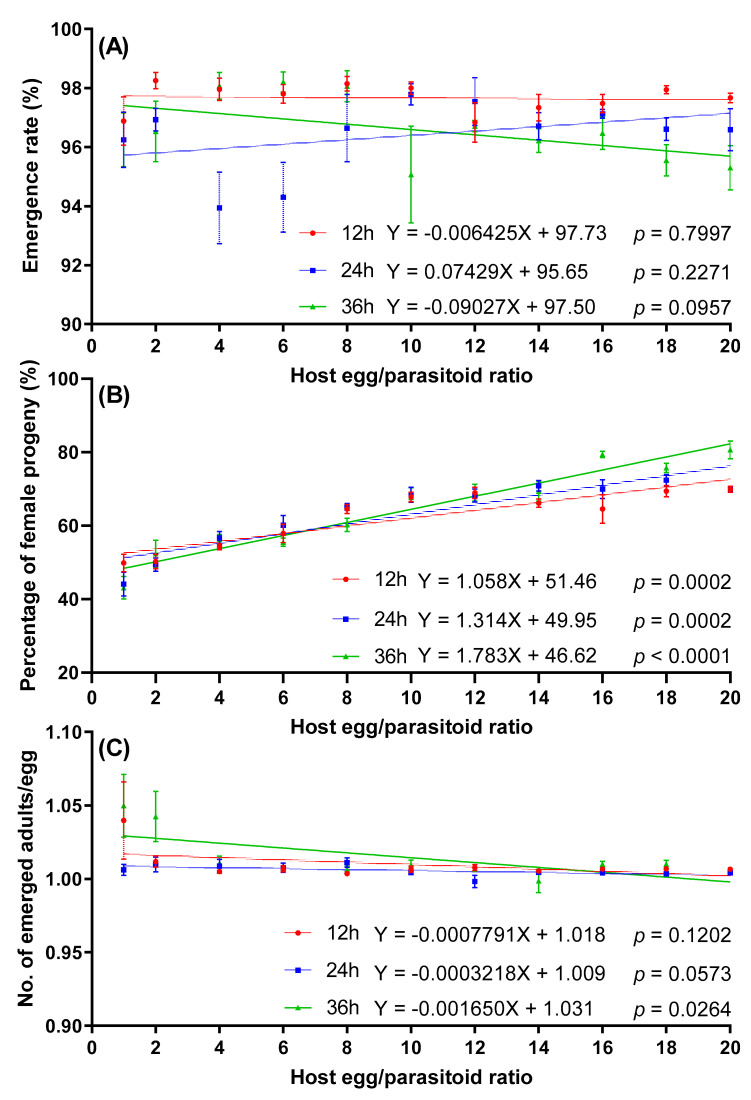
Effect of host egg:parasitoid ratio on emergence rate (**A**), percentage of female progeny (**B**), and number of emerged adults per egg (**C**) of *T. remus* on *S. litura* eggs.

No significant linear relationship was observed between the emergence rate and host egg:parasitoid ratio at any exposure time. In each treatment, the emergence rate of progeny was higher than 94.30% (Figure 5A).

The interaction between exposure time and host egg:parasitoid ratio had significant effects on the percentage of female progeny (Table 2). The percentage of female progeny significantly increased with an increased host egg:parasitoid ratio at any exposure time. The lowest percentage of female progeny was at the 1:1 host egg:parasitoid ratio, with mean percentage female for 12, 24, and 36 h of exposure time at 49.84%, 44.12%, and 43.13%, respectively (Figure 5B). No significant linear regression was observed between the number of emerged adults per egg and host egg:parasitoid ratio at exposure times of 12 or 24 h. However, there was a significant negative correlation at 36 h of exposure time (Figure 5C).

## 4. Discussion

Our experiments tested the effect of photoperiod, exposure time, and host egg:parasitoid ratio on survival, offspring fitness, and reproductive potential of *T. remus* reared on *S. litura* eggs. Equally high reproductive potential was observed under the photoperiods of 12:12, 16:8, 20:4, and 24:0 L:D. The optimal host egg:parasitoid ratio was 14–20:1, and the optimal exposure time was 24 h. These are the ideal conditions of photoperiod, exposure time, and host egg:parasitoid ratio for mass-rearing this strain of *T. remus* using *S. litura* eggs as the alternative host.

Despite being a promising candidate agent in the biocontrol of *S. frugiperda*, there are few studies on the physiological and reproductive performance of *T. remus* in China, despite this information being critical for improving mass-rearing efficiency in a laboratory. This study, to the best of our knowledge, is the first to systematically measure the effects of photoperiod on *T. remus* reared on *S. litura* eggs under controlled conditions. Our results revealed that the biological characteristics of *T. remus* on *S. litura* eggs depend closely on photoperiod: The all-dark photoperiodic condition caused the worst performance, and increasing illumination time positively affected the total number of parasitized eggs. We observed that *T. remus* frequently rested in darkness, suggesting that this species is mainly active during daytime. The increase in parasitism observed under long illumination suggests that the day-active parasitoids are time-limited rather than egg-limited [42]. Higher parasitism capacity for *T. remus* on *S. frugiperda* eggs, for *T. podisi* on *Euschistus heros* (Fabricius) eggs, and for *T*. *pretiosum* on *Anagasta kuehniella* (Zeller) eggs were also recorded during light compared to dark [43]. A prior study on *Venturia canescens* (Gravenhorst) observed that this species moved slowly, hardly tried flight, and exhibited abnormal activities under increased darkness conditions [44]. It is generally accepted that parasitoids are more active during daytime, mainly for parasitism, migration, and escaping from predators [45]. However, this positive relationship between photoperiod and parasitism cannot extend to all parasitoids: Okzan [45] found that *V. canescens* increased their egg load under continuous darkness and Mbata et al. [46] found photoperiod had no significant impact on the oviposition of *Plodia interpunctella* (Hübner). In addition, reports indicated that continuous daytime decreased the lifespan of *V. canescens*. This might be because the parasitoids reduced activity under darkness, which would save energy to support their survival [44]. In the present study, the shortest *T. remus* female longevity was also observed with a 24-h light photoperiod, suggesting that increased activity and parasitism during extreme illumination comes at the cost of decreased longevity. However, the effect of photoperiod on the longevity of females was not linear, with a marked decrease at 12 h light. The reason and mechanism for this observed sensitivity to the 12:12 L:D photoperiod need to be further explored.

In addition to photoperiod as studied in the current study, the light intensity associated with it also had a significant impact on parasitoids [47]. As reported by Hu et al. [47], long-day photoperiods and high light intensity produced more winged female *S. pupariae* progeny, and their interactions significantly impacted the developmental time and the degree of phenotypic partitioning of the progeny. Light intensity may affect flight initiation and orientation of the parasitoids, which affects their ability to find hosts [48,49]. Therefore, future research should pay attention to the role of light intensity in the mass-rearing and application of *T. remus*.

Understanding the relationship between photoperiod and biological characteristics of insects helps optimize insect rearing [50]. Musolin and Saulich [51] found that the growth rate of Orthoptera was retarded by short photoperiods and accelerated by long photoperiods. The current study observed a similar phenomenon, where the mean generation time was shortest under the photoperiod of 24L:0D. Exceptions exist: Hu et al. [47] found *S. pupariae* developed faster under prolonged darkness.

In terms of oviposition rhythm, research suggests the pre-oviposition period, namely egg retention, is prolonged with shorter photoperiods, perhaps due to reproductive diapause [52]. However, a pre-oviposition period was not observed in this study. The percentage of female progeny was highest for eggs laid on the first day of emergence regardless of photoperiod, and then gradually decreased. Diapause or dormancy for most arthropods is triggered by the interaction of colder temperatures with shorter photoperiods [53]. Malaquias et al. [54] illustrated that photoperiod is a vital ecological factor regulating oogenesis via the neuroendocrine system. In nature, the physiological and developmental preparations of organisms for seasonal reproduction, dormancy, and migration events generally must be done prior to that season, and day length offers a reliable reference for animals to anticipate seasonal change [55]. Therefore, photoperiodicity can serve as an anticipatory response of organisms to seasonal events and day length cues [53,55]. Generally, the physiology of many insects mainly focuses on reproduction when they are exposed to long-day conditions, whereas short photoperiod induces diapause as the corpora allata are deactivated, thus inhibiting the secretion of juvenile hormone [50]. In future laboratory and field studies, interactions between temperature and photoperiod should be investigated to determine whether *T. remus* undergoes diapause or not, and, if it does, identify the key factors inducing diapause to provide guidance for optimizing production efficiency and long-term cold storage.

The emergence rate and percentage of female progeny of *T. remus* were not impacted by the photoperiod. One possible explanation for these results might be the short longevity of adults, which does not provide enough time for sex determination and regulation mechanisms in response to the photoperiod experienced by the maternal generation [56]. Current study mainly evaluated the fitness of individual females under different photoperiods. However, the mass production of parasitoids usually involves a large quantity of individuals in a limited space. Under this context, local mate competition (LMC) would be triggered by the competition among males for mating access to females. To reduce or avoid this situation, mothers have fewer male offspring, resulting in a female-biased sex ratio. Although this phenomenon is beneficial to enhance their control efficiency in a natural state, we still need to pay attention to the large-scale production in the industry, and further explore the mechanisms behind this phenomenon [57].

From the perspective of reproductive potential, the net reproductive rate, intrinsic rate of increase, and finite rate of increase of *T. remus* under the photoperiods of more than 12 h light were higher than that under the illumination conditions of less than 12 h light. Thus, the daily illumination time for mass-rearing *T. remus* should be at least 12 h.

The amount of host eggs and time available for the parasitoid to parasitize these eggs also affects mass production efficiency and the quality of the final product [58]. In this study, a relatively high host egg:parasitoid ratio (14–20:1) and exposure for a moderate time (24 h) were critical to achieve both a stable parasitism rate and a percentage of female progeny exceeding 60%. When hosts are provided to a group of parasitoids, females may interfere with each other directly through displaying, fighting, and obstructing other individuals’ access to the hosts; or indirectly by modifying host exploitation strategies such as sex allocation, superparasitism, and clutch size decisions [33,59,60]. In this study, the host egg:parasitoid ratio affected the parasitism rate differently depending on exposure time. The parasitism rate decreased with an increasing host egg:parasitoid ratio at 12 and 36 h of exposure time, but increased at 24 h. Short exposure time (12 h) may not be enough for the females to parasitize oversupplied eggs, causing inefficient use of host eggs and increasing production costs [61]. Long exposure time (36 h) may result in superparasitism due to a shortage of unparasitized hosts, decreasing the successful development and emergence rate [61]. Although previous studies demonstrated that female *T. remus* typically lay a single egg into each host egg, superparasitisim has been recorded in the laboratory. Parasitoid parents would lay more eggs in one host egg when host eggs are scarce or seldom encountered, rather than spending time and energy attempting to find new host eggs [22,62]. However, the limited nutrients within host eggs are usually only enough for one wasp larva to complete its development. This fierce competition for nutrients causes cannibalism and increases parasitoid larval mortality [19]. Similarly, a previous study found exposure time significantly impacted the parasitism rate of *Dirhinus giffardii* (Silvestri) on pupae of *Zeugodacus cucurbitae* (Coquillett) and *Bactrocera dorsalis* (Hendel), with the highest parasitism rate observed after exposure for 48 h, followed by 72 and 24 h [63].

Too many male progenies during mass-rearing reduces production effectiveness [64]. In all our tests, the percentage of female progeny increased with an increasing host egg:parasitoid ratio. The lowest percentage of female progeny was observed at a 1:1 host egg:parasitoid ratio, possibly due to superparasitism, or weak/lack of LMC. In order to maximize offspring fitness, female parasitoids determine and allocate an optimal clutch size. However, under superparasitism conditions, a larger clutch size decreases the fitness of the progeny [62,65]. Another possible reason is that a female parasitoid can maximize her fitness by reducing the number of sons and increasing the number of daughters under high densities of conspecifics [66,67]. Thus, if unable to find more eggs due to a shortage, parasitoids may unintentionally produce more males. A similar study found that, when the parasitoid host ratio was high, *Tetrastichus planipennisi* (Yang) not only produced more male progenies, but also had offspring of lower quality, with a smaller body size and shorter ovipositors [68]. Superparasitism was not clearly confirmed by that paper, but they found that multiple female wasps usually gathered around a single host and seemingly oviposited in the same host [68]. In our study, no significant linear regression was observed between the number of emerged adults per egg and host egg:parasitoid ratio at exposure time of 12 and 24 h, but the significant negative correlation was recorded at 36 h of exposure time. Reduced offspring quantities per host when the exposure time was too long in this study suggest that host resources had been overexploited, resulting in superparasitism. Alternatively, Montoya et al. [69] suggested superparasitism might be an effective reproductive strategy for *Diachasmimorpha longicaudata* (Ashmead), as superparasitism ranging from moderate to high levels lead to more female progenies, ensuring the survival of at least one female larva [70]. In addition, host size and parasitoid densities also strongly affect percentage of the female progeny [69].

## 5. Conclusions

In our experiments, increased photoperiod, moderate exposure time, and host egg:parasitoid ratio improved the mass-rearing efficiency of *T. remus*. We advocate using more than 12 h of light, 24-h exposure time, and a 14–20:1 host egg:parasitoid ratio for rearing *T. remus* on *S. litura* eggs. These findings will help promote the successful, large-scale rearing of *T. remus* for use against *S. frugiperda* in China, though more information is needed to optimize the mass-rearing system further.

## Figures and Tables

**Table 1:** Life table parameters of *T. remus* on *S. litura* eggs under different photoperiods (*n* = 5).

Photoperiod (Light-Dark)	Life Table Parameters			
	Net Reproductive Rate (*R*_0_)	Intrinsic Rate of Increase (*r_m_*)	Finite Rate of Increase (*λ*)	Mean Generation Time (*T*)
0–24	55.16 ± 2.93 d	0.354 ± 0.021 b	1.424 ± 0.029 b	11.5 ± 0.7 ab
4–20	97.81 ± 3.26 bc	0.383 ± 0.016 b	1.470 ± 0.023 b	12.0 ± 0.5 ab
8–16	88.40 ± 3.86 c	0.359 ± 0.020 b	1.432 ± 0.029 b	12.6 ± 0.6 a
12–12	117.96 ± 6.75 a	0.414 ± 0.013 ab	1.514 ± 0.019 ab	11.5 ± 0.3 ab
16–8	116.08 ± 3.82 a	0.395 ± 0.013 ab	1.484 ± 0.019 ab	12.1 ± 0.3 ab
20–4	107.96 ± 3.33 ab	0.423 ± 0.019 ab	1.526 ± 0.029 ab	11.1 ± 0.5 ab
24–0	125.20 ± 1.89 a	0.463 ± 0.007 a	1.590 ± 0.010 a	10.4 ± 0.1 b
F	36.631	5.524	5.944	2.167
df	6, 28	6, 28	6, 28	6, 28
*p*	<0.0001	0.001	<0.0001	0.077

Data are expressed as mean ± SE. Data in a column followed by different letters are significantly different at α = 0.05 (Tukey test).

**Table 2:** Results from ANOVA analysis on the effects of exposure time, host egg:parasitoid ratio, and their interactions on the biological parameters of *T. remus* on *S. litura* eggs.

Parameters	Source	df	F	*p*
Parasitism rate (%)	ET	2	41.753	<0.0001
	HPR	10	4.422	<0.0001
	ET × HPR	20	2.364	0.002
	Error	132		
Emergence rate (%)	ET	2	11.101	<0.0001
	HPR	10	0.981	0.463
	ET × HPR	20	2.710	<0.0001
	Error	132		
Percentage of female progeny (%)	ET	2	5.117	0.007
	HPR	10	63.486	<0.0001
	ET × HPR	20	3.297	<0.0001
	Error	132		
No. of emerged adults/egg	ET	2	4.042	0.02
	HPR	10	4.48	<0.0001
	ET × HPR	20	1.458	0.107
	Error	132		

ET = exposure time, HPR = host egg:parasitoid ratio.

## Data Availability

Data can be provided on request from the lead author.
